# Bioactives and Inflammation

**DOI:** 10.3390/cimb45070368

**Published:** 2023-07-13

**Authors:** Guan-Ting Liu, Chan-Yen Kuo

**Affiliations:** Department of Research, Taipei Tzu Chi Hospital, Buddhist Tzu Chi Medical Foundation, New Taipei City 23142, Taiwan; mochabear19@gmail.com

Inflammation is one of the body’s most complex physiological defense mechanisms against harmful substances. Prolonged, excessive inflammatory responses can cause long-term damage to the body, including chronic health problems. The effects of natural bioactives on inflammation and disease have been extensively researched, with studies demonstrating that bioactives can minimize the risk of inflammatory disease, while enhancing well-being and patients’ quality of life. This Special Issue includes 22 articles from a great variety of international submissions. The purpose of this collection is to facilitate the acquisition of new knowledge and to stimulate the wider application of available tools in future research. A number of these articles provide an overview of anti-inflammatory compounds, natural products, synthesized compounds, and novel mechanisms and targets related to anti-inflammatory compounds.

Lee et al. demonstrated that macrophages play a critical role in the regulation of the inflammatory response during the progression of periodontal disease. They found that *Magnolia kobus* extract (MKE) had an anti-inflammatory effect on *Porphyromonas gingivalis* lipopolysaccharide (LPS)-induced proinflammatory cytokines, including interleukin-1β (IL-1β), IL-6, tumor necrosis factor-α (TNF-α), matrix metalloproteinases (MMP), high mobility group box 1 (HMGB1), intercellular adhesion molecule (ICAM-1), and vascular cell adhesion protein 1 (VCAM-1) expression in human gingival fibroblast (HGF-1) cells. MKE was also found to regulate osteoclastogenesis via the TLR4/NF-κB pathway in RANKL-stimulated RAW264.7 cells. The MKE treatment also reduced extracellular matrix (ECM) degradation by regulating MMP and acting as a tissue inhibitor of metalloproteinase (TIMP) expression [1].

Cañellas-Santos et al. investigated cell freeze-dried lysate from *Camellia sinensis* and were able to minimize the production of IL-6, IL-8, TNF-α, chemokine (C-X-C motif) ligand-1 (CXCL-1), and the nuclear translocation of the nuclear factor kappa light chain enhancer of activated B cells (NF-κB) in *Cutibacterium acnes*-stimulated human keratinocytes. Moreover, the lysate displayed no bactericidal effects, but decreased biofilm formation, lipase activity, and autoinducer-2 (AI-2), a signaling molecule associated with quorum quenching [2].

Yadav et al. reported that serratiopeptidase (SRP), which originates from microorganisms living in the silkworm (*Bombyx mori*), significantly decreased vascular inflammation by inhibiting the production of proinflammatory cytokines such as IL-2, IL-1, IL-6, and TNF-α by LPS in the aortic tissue of BALB/c mice by inhibiting their production. Moreover, SRP reduced the expression of monocyte chemoattractant protein-1 (MCP-1) and its activity in mice aortas, and inhibited LPS-induced oxidative stress [3].

As a hydrophilic carotenoid pigment, crocin is found in *Crocus sativus* and *Gardenia jasminoides* stigmas. Sangare et al. proposed that crocin inhibits IL-1β secretion, caspase-1 cleavage, Gasdermin D (GSDMD) cleavage, and pyroptosis in J774A.1 mouse macrophages. It also inhibited the attenuated oligomerization of apoptosis-associated speck-like proteins containing a caspase recruitment domain (ASC) and the production of mitochondrial reactive oxygen species (mtROS). Furthermore, crocin inhibited IL-1β and IL-18 production, neutrophil recruitment, and NLRP3 inflammasome activity in vivo following MSU-induced peritonitis [4]. 

Kweon et al. revealed that aquatic *Aloe saponaria* (AAS) extract displays significant antioxidant activities and can greatly regulate the inflammatory response. In dextran sulfate sodium (DSS)-induced ulcerative colitis mice, AAS extract significantly restored the phosphatidylinositol-3-kinase/serine-threonine protein kinase (PI3K/Akt) and phosphatidylinositol-3-kinase/serine-threonine protein kinase/Akt (PI3K/Akt) signaling pathways [5]. 

Bertuccio et al. found that, in the presence of LPS, Ozoile inhibited the expression of TNF-α, IL-1β, ZO-1, CLDN1, NOS2 and MMP-2 and enhanced the expression of NF-E2-related factor 2 (Nrf2) and superoxide dismutase 2 (SOD2) antioxidant proteins in a human colorectal adenocarcinoma HT-29 cell line. Ozoile-treated human leukemia monocytic THP-1 cells prevented the induction of LPS, IL-1 and ICAM. Additional studies are required in animal models in order to verify Ozoile’s potential as an adjuvant therapy in the treatment of intestinal inflammatory conditions [6]. 

*Carica papaya* leaf extraction exhibits anti-inflammatory and anti-hyperglycemic activities by modulating inflammatory molecules, such IL-1β, IL-6, TNF-α, and inhibiting nuclear factor kappa-B kinase subunit beta (IKKβ) and the mammalian target of rapamycin (mTOR); meanwhile, it restores normal levels of antioxidant enzymes, oxidative stress markers, and gene expression analysis in type-2 diabetes experimental rats fed with a high-fat diet [7].

Kuramoto et al. reported that caffeic acid phenethyl ester (CAPE), a physiologically active ingredient found in propolis, inhibits inflammation in human dental pulp cells (HDPCs). Their results indicated that CAPE stimulated the phosphorylation of mitogen-activated protein kinases (MAPK), extracellular signal-regulated kinases (ERK), and stress-activated protein kinase/c-Jun N-terminal kinases (SAP/JNK). They also demonstrated that CAPE inhibited the production of CXCL10 by Pam3CSK4- and TNF-α-stimulated HDPCs [8]. 

Cho et al. found *Sargassum horneri* (*Turner*) *C. Agardh* extract to be nontoxic to BV-2 microglia cells. Furthermore, it significantly lowered the LPS-induced production of nitric oxide (NO) and reduced the activation of microglia induced by LPS via inducible nitric oxide synthase (iNOS), cyclooxygenase-2 (COX-2), and cytokine production. *S. horneri* displayed anti-inflammatory effects, in part, by inhibiting the phosphorylation of both p38 MAPK and NF-κB. Also, *S. horneri* decreased the activation of astrocyte and microglia in mice brains challenged with LPS [9]. 

Alkhalifah et al. found that cardamom extract modulated the Nrf2/heme oxygenase-1 (HO-1)/ AD(P)H-quinone oxidoreductase 1 (NQO-1) pathway to alleviate oxidative stress by regulating the activities of SOD, catalase, glutathione peroxidase (GSH-Px), glutathione reductase (GSH-R), and the inflammatory response by modulating IL-1β, IL-6, TNF-α and NF-κB. It also modulated apoptosis by mediating the expression of caspase 3 and 9. In addition, Bax was produced as a result of acetaminophen-induced hepatic toxicity [10].

Camargo et al. established an experimental model of non-alcoholic steatohepatitis using western diet (WD)-fed ApoE knockout male mice. The WD and high-fat diet (HFD) mice displayed greater levels of whole-body fat than the normally fed (Chow) mice. They also had greater glucose intolerance, and increased hepatic triglycerides, plasma alanine transaminase, and plasma aspartate aminotransferase (AST) levels compared to the Chow mice. The WD mice had higher levels of galectin-3 expression compared to the Chow or HFD mice, and higher levels of plasma cholesterol compared to the Chow mice. The HFD and WD mice experienced increased F4/80 expression and hepatic fibrosis. A higher level of plasma MCP-1 was also detected in the WD mice. The inflammatory markers found in the liver of the WD mice were elevated, including TNF-α, MCP-1, IL-6 and interferon gamma (IFN-γ) [11]. 

Tsai et al. demonstrated that the compound 4-(phenylsulfanyl) butan-2-one (4-PSB-2) has anti-inflammatory and neuroprotective properties. They found that 4-PSB-2 suppressed the production of CC chemokine ligand-1 (CCL-1) in response to LPS. Additionally, 4-PSB-2 suppressed the actions of the mitogen-activated protein kinase and NF-κB pathways intracellularly. CCL-1 production was also suppressed by the epigenetic regulation of 4-PSB-2 via the downregulation of histone H3 and H4 acetylation [12]. 

Fatty acid glucoside (FAG) isolated from *Ficus benghalensis*, a new epidermal growth factor receptor (EGFR) inhibitor, exerted anti-inflammatory activities by inhibiting the AKT/PI3K pathways. Upon LPS treatment, non-cytotoxic concentrations of FAG enhanced NO production and iNOS activity, inhibited COX-1 and COX-2 activities, and decreased prostaglandin E2 (PGE2) levels in RAW 264.7 cells. There was a decreased expression of TNF-α, IL-6, PGE2, EGFR, Akt, and PI3K in the presence of FAG with a directly high infinity for EGFR, according to the molecular docking study [13]. 

Wasabi (*Wasabia japonica*) contains 6-(Methylsulfinyl) hexyl isothiocyanate (6-MSITC), a bioactive substance that inhibits the production of IL-6 and CXCL10 in TR146 cells, which are human oral epithelial cells. Additionally, 6-MSITC inhibited the activation of the STAT3, NF-κB, and p70S6 kinase (p70S6K)-S6 ribosomal protein (S6) pathways in TNF-α-stimulated TR146 cells. A number of STAT3 and NF-κB inhibitors have been shown to inhibit the production of IL-6 and CXCL10 by TR146 cells treated with TNF-α [14]. 

As demonstrated by Ha et al., Apigetrin, a glycosidic flavonoid derived from *Teucrium gnaphaloides*, inhibits iNOS and COX-2 expression in skeletal muscle cells (L6) in a nontoxic, dose-dependent manner. Apigetrin inhibited the LPS-induced phosphorylation of p65 and IκB-α. NF-κB modulated several proinflammatory genes and regulated the inflammatory response. It is also noteworthy that the MAPK signaling pathway comprises ERK, JNK, and p38, which are crucial to the production of cytokines and the downstream signaling events leading to inflammation. In LPS-stimulated cells, Apigetrin significantly decreased the phosphorylations of JNK and p38, but not ERK phosphorylations. Taken together, these findings suggest that Apigetrin minimizes the inflammation induced by LPS in L6 skeletal muscle cells via the NF-κB/MAPK signaling pathways [15].

Gunes-Bayir et al. reported that rheumatoid arthritis (RA) is thought to be pathophysiologically influenced by genetic and environmental factors. Genetically predisposed individuals can experience an enhanced inflammatory response due to their diet. The anti-inflammatory and antioxidant properties of specific diets alleviate the symptoms of RA. Moreover, several types of natural compounds may be effective for managing RA, including flavonoids, polyphenols, carotenoids, and alkaloids, which possess antioxidant properties, as well as probiotics. Using PubMed, ScienceDirect, and Google Scholar databases, this article examined the nutritional approaches employed in order to prevent or extenuate disease progression. Evidence indicates that Mediterranean and vegan diets may facilitate the healing of RA, as fruits, vegetables, grains, nuts, and seeds are rich in dietary fiber, antioxidants, and anti-inflammatory compounds. Vegans should be closely monitored for nutrient intake, as the Mediterranean diet includes additional animal nutrients, such as omega-3 polyunsaturated fatty acids, which are found in fish and seafood. Several RA patients have reported receiving benefits from calorie-restricted and intermittent fasting diets. Solid evidence-based recommendations or guidelines can only be established if additional studies are conducted. Nutrition needs to be routinely addressed using a dietary approach that considers anti-inflammatory properties in order to facilitate the management of RA [16].

Based on the review article by Starikova et al., L-arginine (Arg) has been demonstrated to be an inflammatory modulator in various pathological conditions besides its well-described role in cardiovascular disease. The immune response can be regulated by targeting L-Arg metabolic pathways. However, their application is limited because the enzymes that hydrolyze L-Arg perform homeostatic functions. eNOS must be present to produce NO. Ammonia detoxification and T cell function are regulated by arginase, a key enzyme in the urea cycle. L-Arg increases bioavailability in order to stimulate the immune cell defense mechanisms but ultimately aids the tumor or pathogen. A reduction in amino acid bioavailability compromises immune function. The increased bioavailability of L-Arg for NOS is accompanied by an increase in arginase activity. Supplementing or depleting L-Arg adversely affects the dysfunctional vascular endothelium. Understanding the L-Arg metabolic pathways in specific pathological conditions is essential to treating the pathology. Therefore, discovering a therapy that regulates the metabolism of L-Arg remains difficult [17]. 

Black cumin, also known as *Nigella sativa* L. (family Ranunculaceae), has been employed in cuisine for thousands of years. Food and medicine benefit from its health-promoting properties. Thymoquinone (TQ) is the principal active compound in black cumin extract, which has hypoglycemic, hypolipemic, and hepatoprotective properties. TQ also has immunomodulatory and anticancer effects, but its contribution to cancer prevention is unknown, according to in vitro tests. Incorporating this plant compound into the diet can be an effective prophylactic and therapeutic measure. However, its potential interactions with drugs that are used to treat chronic diseases must be monitored [18]. 

Garlic (*Allium sativum*) has been employed for centuries as a traditional medicine and for culinary purposes; it has a variety of health benefits, including antioxidant, anti-inflammatory, antitumor, anti-obesity, anti-diabetic, anti-allergic, cardioprotective, and hepatoprotective effects. One of the most important garlic extracts is aged garlic extract (AGE). Allicin, which is found naturally in raw garlic, is a sour, odorous, and irritating substance that has significant therapeutic benefits when converted into a stable and safe compound, such as S-allylcysteine; this compound has antioxidant, anti-inflammatory, and immunomodulatory properties. AGE is a promising candidate for regulating cytokine secretion, promoting phagocytosis, and activating macrophages. AGE has been suggested for application as a treatment for chronic inflammatory bowel disease due to the significant role that immune dysfunction plays in many diseases [19]. 

Prokineticins (PKs) are a group of secreted peptides that possess chemotactic and immunomodulatory properties. One PK molecule binds to two G protein-coupled receptors. In addition to angiogenesis and neurogenesis, PK receptor 2 (PKR2) plays a role in hematopoiesis, hypothalamic hormone secretion, circadian rhythms, and feeding and drinking. The dysregulation of this system leads to an inflammatory response, resulting in cancer, pain, neuroinflammation, and neurodegenerative diseases, such as Alzheimer’s and Parkinson’s diseases. Upregulating PK2/PKRs with a PKR antagonist is an effective method by which to treat neuroinflammatory diseases. PKR agonists have been developed to modulate the PK system [20]. 

In this review, Scanu et al. provide an overview of compounds that promote hyperuricemia and gout, as well as inflammation. Monosodium urate crystals are deposited in the joints of men and cause gout. This disease mainly occurs when increased hyperuricemia is present. Hyperuricemia also increases inflammation, enhancing the risk of comorbid diseases such as cardiovascular disease. Chronic inflammatory and immune conditions have been linked to bioactive compounds. These molecules affect gout and hyperuricemia differently. Hyperuricemia and monosodium urate crystal deposits are treated using medications that reduce the serum uric acid concentration, making them a valuable adjunct to pharmacological therapy and disease prevention [21].

Bilberries comprise phenolic acids, organic acids, anthocyanins, coumarins, flavonols, flavanols, tannins, terpenoids, and volatile chemicals. Based on data obtained from cell and animal model studies, bilberries possess anti-inflammatory effects; they reduce the expression of TNF, IL-6, NO synthases, and cyclooxygenases, as well as alter the NF-κB and Janus kinase (JAK)-dependent pathways. Supplementing the diet with bilberries as fruits (frozen, processed, and whole), juices, and anthocyanins could reduce inflammatory markers in patients with metabolic disorders. Thus, bilberries may be useful for preventing and treating chronic inflammatory diseases [22].
